# Toxin YafQ Reduces *Escherichia coli* Growth at Low Temperatures

**DOI:** 10.1371/journal.pone.0161577

**Published:** 2016-08-24

**Authors:** Yueju Zhao, Michael J. McAnulty, Thomas K. Wood

**Affiliations:** 1 Institute of Food Science and Technology, Chinese Academy of Agricultural Sciences, Beijing, 100193, P. R. China; 2 Key Laboratory of Agro-products Processing, Ministry of Agriculture, Beijing, 100193, P. R. China; 3 Department of Chemical Engineering, Pennsylvania State University, University Park, Pennsylvania, 16802-4400, United States of America; 4 Department of Biochemistry and Molecular Biology, Pennsylvania State University, University Park, Pennsylvania, 16802-4400, United States of America; Centre National de la Recherche Scientifique, Aix-Marseille Université, FRANCE

## Abstract

Toxin/antitoxin (TA) systems reduce metabolism under stress; for example, toxin YafQ of the YafQ/DinJ *Escherichia coli* TA system reduces growth by cleaving transcripts with in-frame 5’-AAA-G/A-3’ sites, and antitoxin DinJ is a global regulator that represses its locus as well as controls levels of the stationary sigma factor RpoS. Here we investigated the influence on cell growth at various temperatures and found that deletion of the antitoxin gene, *dinJ*, resulted in both reduced metabolism and slower growth at 18°C but not at 37°C. The reduction in growth could be complemented by producing DinJ from a plasmid. Using a transposon screen to reverse the effect of the absence of DinJ, two mutations were found that inactivated the toxin YafQ; hence, the toxin caused the slower growth only at low temperatures rather than DinJ acting as a global regulator. Corroborating this result, a clean deletion of *yafQ* in the Δ*dinJ* Δ*Km*^*R*^ strain restored both metabolism and growth at 18°C. In addition, production of YafQ was more toxic at 18°C compared to 37°C. Furthermore, by overproducing all the *E*. *coli* proteins, the global transcription repressor Mlc was found that counteracts YafQ toxicity only at 18°C. Therefore, YafQ is more effective at reducing metabolism at low temperatures, and Mlc is its putative target.

## Introduction

Most Bacteria and Archaea contain toxin/antitoxin (TA) systems [1–3] which reduce cell growth to enable the cells to cope with stress [4]. For example, the MqsR/MqsA TA system enables the cell to withstand oxidative [5] and bile acid stress in the gastrointestinal tract [6]. Usually the genes for TA systems occur in pairs, and many antitoxins regulate the TA locus [7]. In addition, some antitoxins such as MqsA of the MqsR/MqsA TA system are intertwined with the general stress response by regulating other loci including levels of the stationary phase sigma factor RpoS [5, 8, 9], and some toxins exhibit a general regulatory effect via post-transcriptional differential mRNA decay [10, 11]. In addition to the general stress response, TA systems also have roles in biofilm formation [9, 10, 12, 13] and in inhibiting the propagation of phage [14–16].

In *Escherichia coli*, there are at least 39 TA systems [17–19]. Among them, the YafQ/DinJ TA system has been associated with several physiological roles. YafQ is an endoribonuclease that cleaves mRNA at in-frame 5’-AAA-G/A-3’ sites in conjunction with ribosomes [20]. Specifically, YafQ binds the 70S ribosome at the A site via three surface-exposed patches of basic residues that appear to directly interact with 16SrRNA, and YafQ residues H50, H63, D67, and H87 participate in acid-base catalysis during mRNA hydrolysis [21]. Its antitoxin is DinJ [22], and like the *mqsRA* locus [23], the *dinJ-yafQ* locus is not subject to conditional cooperativity [24], a form of regulation in which the binding of the first toxin to the antitoxin represses the TA locus whereas additional toxin molecules induce transcription of the locus [25]. DinJ binding to DNA as a dimer is facilitated by its N-terminal ribbon-helix-helix motif, and its C terminus binds YafQ as a heterotetramer [24, 26]. Also, the SOS regulator LexA binds the *dinJ* promoter suggesting a link of expression of this operon after DNA damage [24] although experimentally this has not been seen [27, 28].

The physiological roles of the YafQ/DinJ TA system include that it actively participates in the general stress response through the regulation of RpoS by antitoxin DinJ via direct repression of *cspE* [29]; cold-shock protein CspE enhances translation of RpoS mRNA. YafQ and DinJ are also involved in regulating persistence in *E*. *coli*; persister cells evade antibiotics by reducing their metabolism via toxins [30, 31] and are responsible for recurring infections [32]. Inactivation of YafQ reduces the persistence of biofilm cells [33], and *dinJ-yafQ* are induced in persisters [34]. The interspecies [35, 36] and interkingdom signal indole [37] reduces persistence [38, 39], and toxin YafQ increases persistence by reducing indole by cleaving tryptophanase mRNA [38]. Notably, indole is most active as a signal in *E*. *coli* at low temperatures [40]. Critically, aside from erythromycin stress leading to DinJ degradation [29], little is known about the conditions that activate YafQ and make it important for its role in the stress response and persistence.

Since there are few reports of temperature affecting the activity of a TA system [41–44], and since little is understood about what activates toxin YafQ, we explored the effect of temperature on the YafQ/DinJ TA system of *E*. *coli*. We found that the deletion of the gene that encodes antitoxin DinJ reduces metabolism and growth only at low temperature and that the mechanism is due to activation of toxin YafQ at low temperature. In addition, it appears the global transcription repressor Mlc may play a role in regulating YafQ activity.

## Materials and Methods

### Bacterial strains, plasmids, and growth conditions

The bacterial strains and plasmids are listed in Table 1. Lysogeny broth (LB) [45] was used for all the experiments. We used the Keio collection [46] for isogenic mutants and pCA24N [47] for expressing genes in *E*. *coli*. One-step inactivation of the *dinJ* and *yafQ* genes in BW25113 using polymerase chain reaction (PCR) products [48] was used to create the double deletion strain, BW25113 Δ*dinJ* Δ*yafQ* (Table 1). The kanamycin resistance cassette from Δ*dinJ*, Δ*yafQ*, and Δ*dinJ* Δ*yafQ* was removed by using plasmid pCP20 [49]. Gene deletions were verified by DNA sequencing using primers *dinJyafQ*-F(CTGGATTTGGAAGGCTCAC) and *dinJyafQ*-R (CATGGATTGTCGCTGTTGC).

**Table 1:** *E*. *coli* bacterial strains and plasmids used in this study.

Strains	Genotype^a^	Source
BW25113	*rrnB3* Δ*lacZ4787 hsdR514* Δ(*araBAD*)*567* Δ(*rhaBAD*)*568 rph*-*1*	[46]
BW25113 Δ*yafQ*	Δ*yafQ* Ω Km^R^	[46]
BW25113 Δ*yafQ* Δ*Km*^*R*^	Δ*yafQ* ΔKm^R^	this study
BW25113 Δ*dinJ*	Δ*dinJ* Ω Km^R^	[46]
BW25113 Δ*dinJ* Δ*Km*^*R*^	Δ*dinJ* ΔKm^R^	this study
BW25113 Δ*dinJ* Δ*yafQ* Δ*Km*	Δ*dinJ* Δ*yafQ* Ω Km^R^	this study
BW25113 Δ*rpoS*	Δ*rpoS* Ω Km^R^	[46]
BW25113 Δ*mlc*	Δ*mlc* Ω Km^R^	[46]
**Plasmids**		
pCA24N	Cm^R^; *lacI*^q^, pCA24N	[47]
pCA24N-*dinJ*	Cm^R^; *lacI*^q^, pCA24N P_T5-lac_::*dinJ*	[47]
pCA24N-*yafQ*	Cm^R^; *lacI*^q^, pCA24N P_T5-lac_::*yafQ*	[47]
pCA24N-*mlc*	Cm^R^; *lacI*^q^, pCA24N P_T5-lac_::*mlc*	[47]
pCA24N-*yhbU*	Cm^R^; *lacI*^q^, pCA24N P_T5-lac_::*yhbU*	[47]
pCA24N-*sspA*	Cm^R^; *lacI*^q^, pCA24N P_T5-lac_::*sspA*	[47]
pCP20	Ap^R^, Cm^R^; FLP^+^, λ*c*I857^+^, λ*p*_*R*_Rep^ts^	[49]

^a^ Cm^R^ and Km^R^ are chloramphenicol and kanamycin resistance, respectively.

Cell growth was assayed using the turbidity at 600 nm. Kanamycin (50 μg/mL) was used for the Keio mutants, chloramphenicol (30 μg/mL) was used to maintain the pCA24N-based plasmids [47], and ampicillin (100 μg/mL) was used to maintain pCP20.

### Transposon mutant selection for increased growth of the Δ*dinJ* Δ*Km*^*R*^ strain

To identify the protein that caused toxicity at 18°C in the Δ*dinJ* Δ*Km*^*R*^ strain, an EZ-Tn5^™^ <KAN-2>Tnp Transposome^™^ Kit (Epicentre) was used to make the mutant library of BW25113 Δ*dinJ* Δ*Km*^*R*^ according to the manufacturer’s instructions. In brief, 1 μL of transposome was electroporated into 50μL of competent cells (1 μL of TypeOne^™^ Restriction Inhibitor was added into the mixture to increase transduction efficiency). Warm SOC medium (1 mL) was used and the cells were incubated for 37°C for 60 min. The recovered cells (100 μL) were diluted 10-fold and spread on LB plates with kanamycin (50 μg/mL) to enumerate the number of transposon insertion clones. The remaining 900 μL of recovered cells (around 6,000 mutant cells) was inoculated into 20 ml fresh medium and cultured at 18°C for 36 hours. After 36 hr, 100 μL culture was transferred to 5 mL of fresh medium and cultured at 18°C for 36 hours (this was repeated four more times for six total enrichment cultures).

After six rounds of growth to enrich for faster-growing BW25113 Δ*dinJ* Δ*Km*^*R*^ transposon mutants, colonies were formed, and 500 independent colonies were cultured in 0.5 mL of fresh medium in 2 mL microcentrifuge tubes for 24 h at 37°C. One μL of culture was inoculated into 100 μL of fresh medium in 96 well plates (two replicates) and BW25113 *ΔdinJ Δkm*^*R*^ was used for comparison. After culturing at 18°C and 250 rpm for 24h, growth was detected by measuring the turbidity at 600 nm. Colonies that yielded cultures with significant increases in growth (growth ratio greater than 50%) were selected for a second round of screening (in triplicate). The position of the transposon insertion was identified following the method of Ducey and Dyer (http://microgen.ouhsc.edu/forms/Epicentre.pdf) using random amplification of transposon ends (RATE) PCR (primers are listed in Table A in S1 File).

### Metabolic activity assay

Overnight cultures were inoculated in LB medium, grown to a turbidity of 1.0 at 600 nm, and resuspended in IF-10 (BioLog, Hayward, CA, USA). Samples were diluted in IF-10 to a turbidity of 0.07 at 600 nm and further diluted 200-fold (turbidity of 0.00035 at 600 nm) into a medium containing IF-10, BioLog Redox Dye D (BioLog) and rich medium (0.1% yeast extract, 0.2% tryptone, and 0.1% NaCl). Cultures were grown at 37°C, 30°C and 18°C in 96-well microtiter plates (100 μL per well) and metabolic activity was monitored by measuring the optical density at 590 nm, indicating both reduction of tetrazolium dye to formazan [50] and sample turbidity. Experiments were performed with at least two independent cultures.

### Pooled ASKA selection for increased growth of the Δ*dinJ* Δ*Km*^*R*^ strain

To identify proteins that reduce YafQ toxicity in the absence of DinJ at 18°C, we produced each *E*. *coli* protein via the ASKA clone set in BW25113 *ΔdinJ ΔKm*^*R*^ and selected for faster growth through successive cultures. One μL (40 ng) of pooled ASKA (GFP-) plasmid DNA [47] was transformed into 50 μL of BW25113 *ΔdinJ ΔKm*^*R*^ competent cells by electroporation (1 μL of TypeOne^™^ Restriction Inhibitor was added into the mixture to increase transduction efficiency). Warm SOC medium (1 mL) was added and the cells were shaken at 37°C for 60 min. The cells were diluted 10-fold, spread on LB Cm30 plates to calculate the number of transposon insertion clones, and 900 μL was inoculated into 20 ml fresh LB Cm30 media with IPTG (200 μM) and cultured at 18°C for 36 hours. 100 μL was then transferred to 5 mL of fresh LB Cm30 IPTG (200 μM) and cultured at 18°C for another 36 hours. This was repeated 5 more times for a total of six successive cultures.

After six rounds of enrichment for faster growth, 100 μL of the cell suspension was diluted and plated on LB Cm30 plates, and 300 colonies were selected. Each colony was cultured in 0.5 ml fresh LB Cm30 media at 37°C for 24 hours, then 1 μL of each culture was inoculated into 100 μL of LB Cm30 media with IPTG 200 μM in 96 well plates. Each colony was assayed as two replicate wells and BW25113 *ΔdinJ ΔKm*^*R*^/pCA24N was used for comparison. After culturing at 18°C and 250 rpm for 24 h, growth was detected by measuring the turbidity at 600 nm. Colonies that yielded cultures with increased growth (increase growth ratio ≥ 50%) were selected for a second round of screening. 40 μL of overnight cultures of BW25113*ΔdinJ ΔKm*^*R*^/pCA24N and the best colonies obtained by the first round of 96-well plate screening were inoculated into 4 mL of fresh LB Cm30 IPTG 200 μM media. After culturing at 18°C and 250 rpm for 24h, growth was detected by measuring the turbidity. Cultures with increased growth (increase growth ratio ≥ 50%) were selected for plasmid extraction. Primers pCA24N-F (5’-GCCCTTTCGTCTTCACCTCG) and pCA24N-R 5’(GAACAAATCCAGATGGAGTTCTGAGGT) were used to identify genes encoded by pCA24N via sequencing. The plasmids of the positive cultures were re-transformed into 50 μL of BW25113 *ΔdinJ ΔKm*^*R*^ competent cells to ensure the phenotype was due to the ASKA plasmid. Overnight cultures of BW25113 *ΔdinJ ΔKm*^*R*^ /pCA24N and the positive cultures obtained by re-transformation were inoculated into 25 mL of fresh LB Cm30 IPTG (200 μM) media at an initial turbidity of 0.05. After culturing at 18°C and 250 rpm for 24 h, growth was detected by turbidity.

### RNA isolation and quantitative, real-time, reverse transcription polymerase chain reaction (qRT-PCR)

To investigate the changes in *yafQ* mRNA levels at 18°C and 37°C, overnight cultures of the wild type strain BW25113 and of BW25113 Δ*dinJ ΔKm*^*R*^ were inoculated into fresh LB at an initial turbidity of 0.05. Cells were collected after 2 hours culture at 37°C or 16 hours at 18°C at a turbidity of 0.7~0.8 for the wild type strain. To avoid RNA degradation, cells were harvested rapidly, cooled using ethanol/dry ice, centrifuged, then the cell pellets flash frozen in ethanol/dry ice. Total RNA was isolated from cells as described previously [51] with a RNeasy Mini Kit (Qiagen, Valencia, CA, USA) using a bead beater (Biospec, Bartlesville, OK, USA) and RNAlater buffer (Applied Biosystems, Foster City, CA, USA) to stabilize the RNA. 100 ng of total RNA was used for qRT-PCR using the Power SYBR Green RNA-to-C_T_1-Step Kit and the StepOne Real-Time PCR System (Applied Biosystems). Primers were designed using Primer-BLAST and are listed in Table B in S1 File. The housekeeping gene *rrsG* was used to normalize the gene expression data. The annealed temperature was 60°C for all the genes in this study.

### Western blot analysis

To investigate the influence of *dinJ* deletion on RpoS at different temperatures, Western blots were performed as described previously [38, 52]. Overnight culture of wild type strain BW25113 and BW25113 Δ*dinJ ΔKm*^*R*^ were inoculated into fresh LB with an initial turbidity of 0.05. Cells were collected after 2 hours of culturing at 37°C or 16 hours at 18°C at a turbidity of 0.7~0.8 for the wild type strain. Cell pellets were processed with 1 mM phenylmethylsulfonyl fluoride and protease inhibitor cocktail (Sigma-Aldrich) and sonicated twice on ice for 15 s. The cell lysate was centrifuged at 13,000 g for 10 min, and the protein concentration of each supernatant was quantified by using the Pierce Protein Assay kit. The same amount of protein (40 μg) was loaded into each well of a 12% SDS-PAGE gel, then transferred to a PVDF membrane, which was then blocked with 4% BSA in TBST (10 mM Tris pH 7.5, 100 mM NaCl, 0.1% Tween 20) for 1 h at room temperature. The Western blots were probed with a 1:2000 dilution of anti-RpoS monoclonal antibody (Neoclone) followed by a 1:20 000 dilution of horseradish peroxidase-conjugated goat anti-mouse secondary antibodies (Millipore).

## Results

### Inactivation of antitoxin DinJ reduces metabolism at 18°C

The PortEco phenotypic database [53] provides scores for growth phenotypes from screening a library of single gene mutants in *E*. *coli* against a panel of 324 different chemical treatments covering 114 unique stresses. Hence, we searched the PortEco database for significant growth changes related to temperature that involved TA systems. The database indicated that a strain lacking the gene that encodes antitoxin DinJ had reduced growth at lower temperatures (score -4.2 at 20°C or at 18°C) [53]. Therefore we explored the impact of the *dinJ* mutation on metabolism at low temperatures. To ensure there were no artifacts, we utilized only strains in which the kanamycin resistance selection marker was deleted so that the only difference was the deletion of the chromosomal copy of the gene. Upon deletion of the kanamycin resistance marker, the promoter regions for the genes were not changed; for example, sequencing of Δ*dinJ* Δ*Km*^*R*^ showed the original promoter and operator region were not altered. In addition, the ribosome binding site for *yafQ* is preserved in Δ*dinJ* Δ*Km*^*R*^ and located in the residual portion of the *dinJ* gene (Fig A in S1 File).

**Fig 1:**
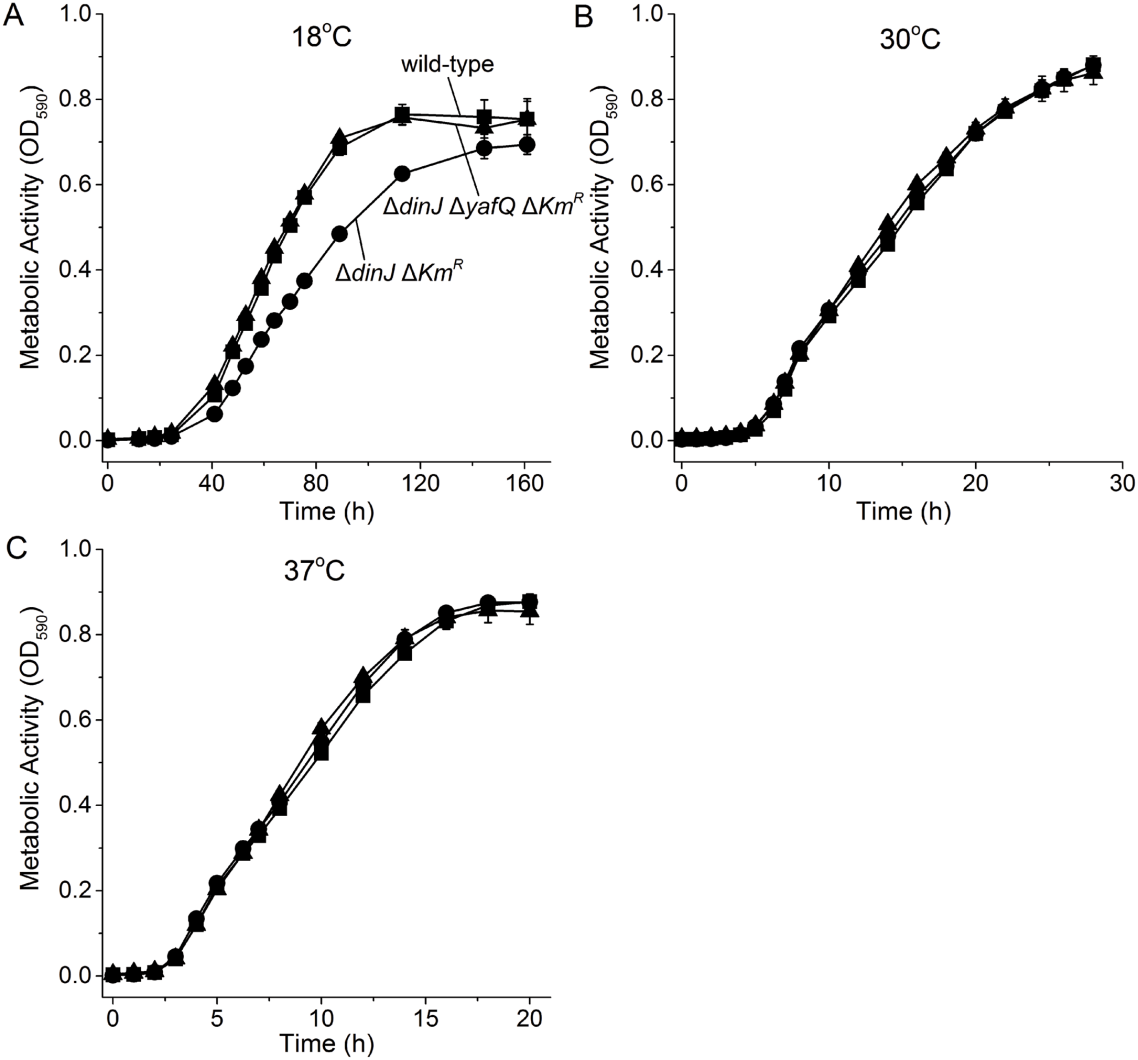
Δ*dinJ* reduces metabolism only at 18°C. Comparison of metabolic activity via the Biolog assay for BW25113 wild-type (■), Δ*dinJ* Δ*Km*^*R*^ (●) and Δ*dinJ* Δ*yafQ* Δ*Km*^*R*^ (▲) at 18°C (**A**), 30°C (**B**), and 37°C (**C**). Data are the average of two independent cultures and one standard deviation is shown.

We first investigated the role the YafQ/DinJ TA system at low temperatures by performing the metabolic activity assay which utilizes tetrazolium dye; tetrazolium dye is reduced to the purple compound formazan due to NADH produced during cellular respiration [50]. We found that deletion of both the toxin and antitoxin had no phenotype whereas deletion of only antitoxin *dinJ* reduced metabolism significantly at 18°C but not at 30°C or 37°C (Fig 1). Therefore, inactivation of antitoxin DinJ reduces cell metabolism only at lower temperatures.

### Inactivation of antitoxin DinJ reduces growth at 18°C

**Fig 2:**
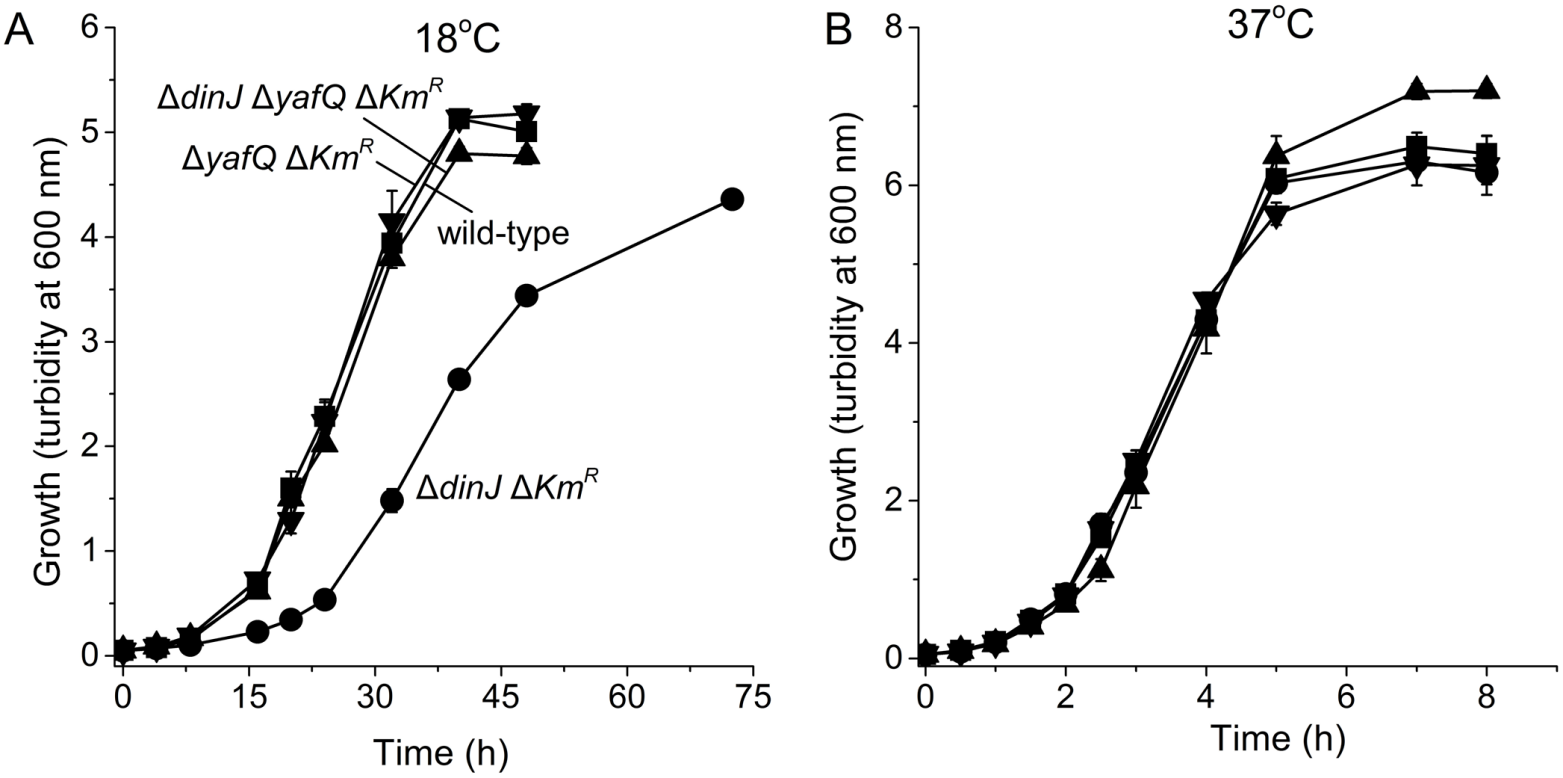
Δ*dinJ* reduces growth only at 18°C. Comparison of growth in LB medium for BW25113 wild-type (■), Δ*dinJ* Δ*Km*^*R*^ (●), Δ*dinJ* Δ*yafQ ΔKm*^*R*^ (▲), and Δ*yafQ* Δ*Km*^*R*^ (▼) at 18°C (**A**) and 37°C (**B**). Data are the average of three independent cultures and one standard deviation is shown.

**Fig 3:**
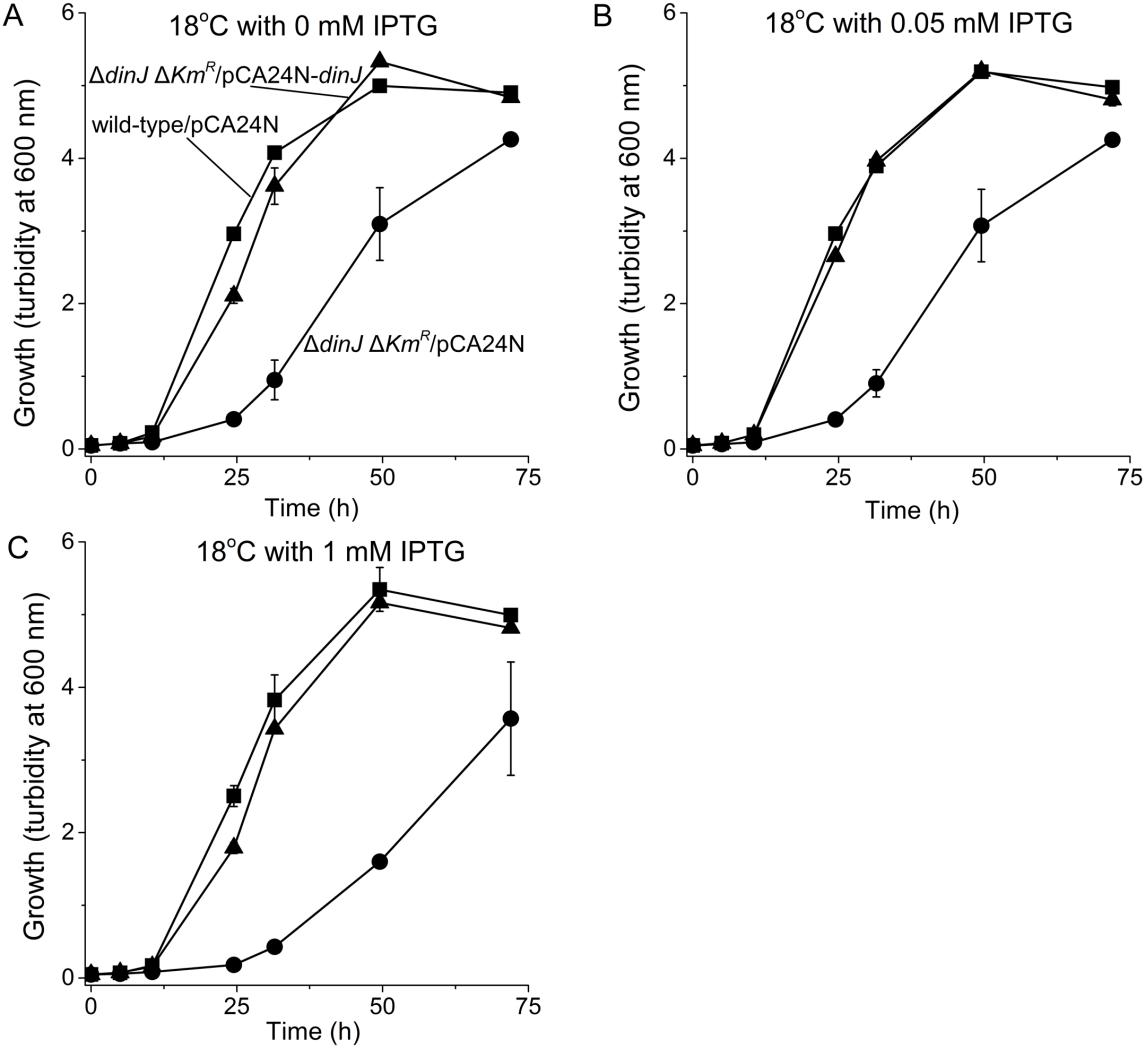
Complementation of the Δ*dinJ* lower growth phenotype at 18°C. Comparison of growth in LB medium for BW25113/pCA24N (■), BW25113 Δ*dinJ* Δ*Km*^*R*^/pCA24N (●) and BW25113 Δ*dinJ* Δ*Km*^*R*^/pCA24N-*dinJ* (▲)at 18°C with IPTG induction of 0 mM IPTG (**A**), 0.05 mM IPTG (**B**), and 1 mM IPTG (**C**). Data are averaged from three independent cultures and one standard deviation is shown.

Since the *dinJ* deletion reduced metabolism only at low temperatures, we checked growth for this strain at 37°C and 18°C. We found that like metabolism, inactivation of DinJ reduced growth only at 18°C (Fig 2). This phenotype could be complemented by producing DinJ from plasmid pCA24N-*dinJ* (Fig 3). Note that low levels of DinJ sufficed to reduce the toxicity since similar results were seen via the leaky P_T5-lac_ promoter at 0 mM IPTG as well as at 0.05 and 1 mM IPTG (Fig 3); some leakiness has been reported for this promoter [47]. As a negative control, the empty plasmid pCA24N did not affect growth of both the wild-type and the *dinJ* strain. Therefore, inactivation of antitoxin DinJ reduces cell growth only at lower temperatures.

### Toxin YafQ reduces metabolism and growth at 18°C

The cause of the reduced metabolism and growth only at low temperatures may be due to DinJ acting as a repressor of loci other than its own by binding at a palindrome (e.g., *cspE* which affects RpoS levels) [29] or due to activation of toxin YafQ. Hence, we randomly inactivated genes via transposon mutagenesis in the slow-growing Δ*dinJ* Δ*Km*^*R*^ strain and selected for mutations that increased growth at 18°C; the transposon mutants were cultured together in LB at 18°C for six successive serial dilution cultures, and the DNA of the fastest growing strains was sequenced to determine where the mutations lie. Approximately 6,000 transposon mutants were formed. Because the wild-type strain grows at least as twice as the *dinJ* deletion strain at 18°C (Fig 2A), after six rounds of enrichment (i.e., six rounds of growth for 36 hr), the faster growing strains with a transposon insertion would be enriched by 33 million fold. We plated the cells after five rounds of enrichment, screened 500 of the transposon mutants in 96 well plates for faster growth, and found 10 mutants that showed a significant increase in growth compared with BW25113 *ΔdinJ ΔKm*^*R*^. After sequencing out from the transposon insertion, along with mutations in *rapA*, *hupA*, *gcvT*, *gcvP*, and *orfE* (Table 2), we found that growth was restored by mutations in the gene which encodes toxin YafQ (mutant found twice). For the two *yafQ* mutants, one had the Tn5 insertion upstream of the start codon (TR2-17), and for the second, the Tn5 insertion (TR2-40) removed the last 22 amino acids of YafQ (from aa 71 to 92). Based on the mutagenesis study by Armalyte J et al. [54], Arg83, His87, and Phe91 are active-site residues of YafQ since the Arg83Ala, His87Ala, and Phe91Ala substitutions abolished mRNA cleavage activity *in vivo*.

**Table 2:** Gene inactivations that restore growth at 18°C.

Mutant	Gene	Function of gene product	Insertion site
TR2-17	*yafQ*	YafQ is a sequence-specific mRNA endoribonuclease	-3/279 (RBS) of *yafQ*
TR2-40	*yafQ*		212/279
TR3-43	*rapA*	RNA polymerase-associated, ATP-dependent RNA translocase; RNA polymerase PTC complex remodeling/recycling factor	268/2907
TR3-17	*rapA*		803/2907
TR3-6	*rapA*		1868/2907
TR4-45	*rapA*		260/2907
TR4-49	*rapA*		1372/2907
TR6-1	*hupA*	Histone-like protein HU-alpha	113/273
TR6-19	*hupA*		1/273
TR3-37	*gcvT*	Aminomethyl transferase	446/1095
TR3-29	*gcvP*	Glycine decarboxylase	347/2874
TR4-53	*orfE*	Pseudogene reconstruction, RNase PH	352/717

Tn*5*-based random mutagenesis was performed with BW25113 Δ*dinJ* ΔKm^R^, and mutations that increase growth were identified by DNA sequencing. Insertion site numbers indicate the position of insertion of Tn*5*; for example, 216/279 means Tn*5* is inserted at the 216nt where position one is start codon.

Corroborating these results, deletion of *yafQ* in the Δ*dinJ* Δ*Km*^*R*^ background restored both metabolism (Fig 1A) and growth (Fig 2A) at 18°C. Also, deletion of the toxin gene *yafQ* (along with the kanamycin marker used to make it) restored the growth seen by the wild-type strain at low temperatures (Fig 2A). These results suggest the reduction in growth seen in the *dinJ* mutant is due to activity of toxin YafQ, rather than repression of other loci by DinJ. Therefore, YafQ reduces growth at low temperatures upon deletion of *dinJ*.

### YafQ is more toxic at lower temperatures

**Fig 4:**
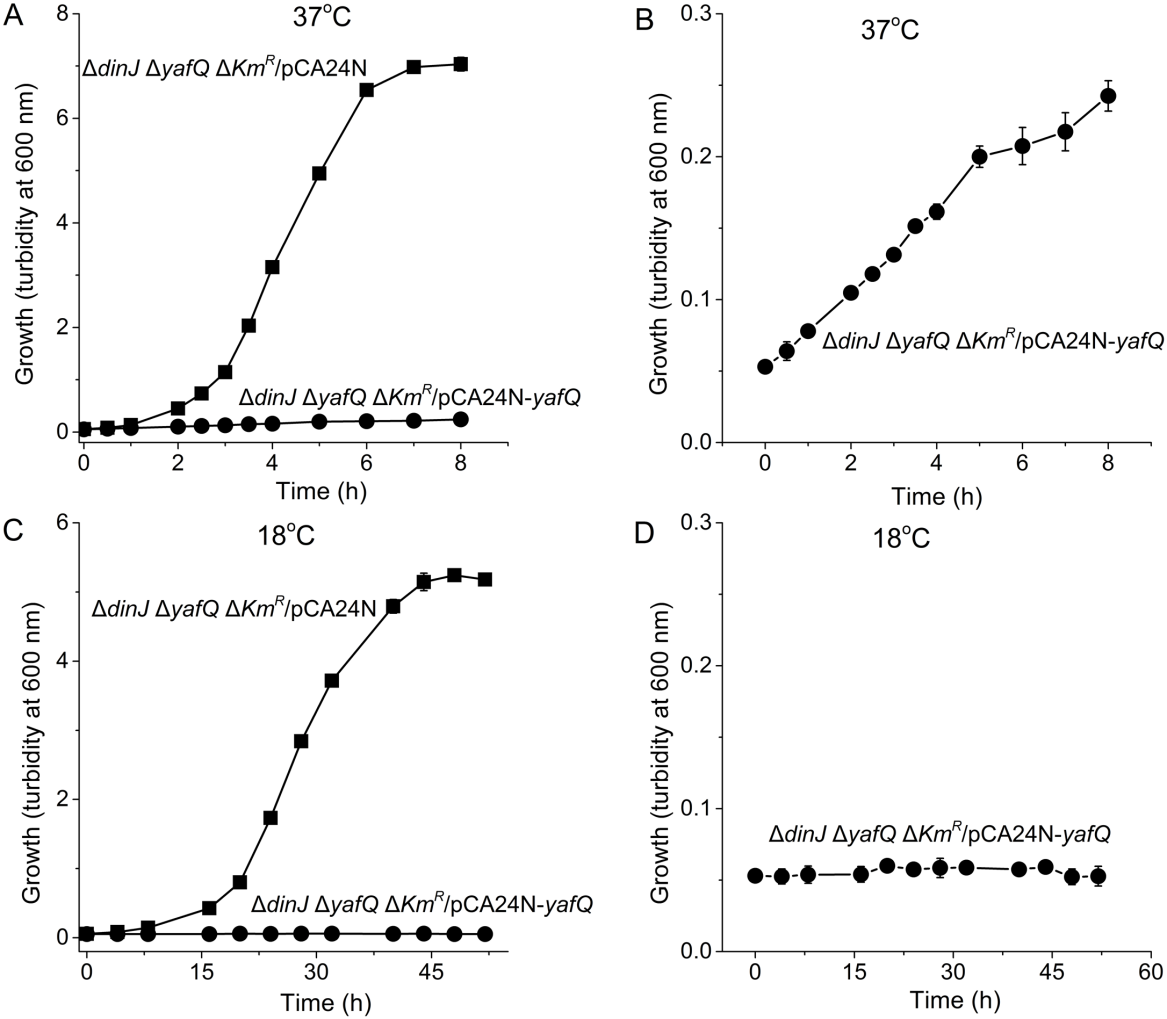
YafQ is more toxic at 18°C. Comparison of growth in LB medium for BW25113 Δ*dinJ* Δ*yafQ ΔKm*^*R*^/pCA24N (■) and Δ*dinJ* Δ*yafQ* Δ*Km*^*R*^/pCA24N-*yafQ* (●) at 37°C (**A**) and 18°C (**C**). Panels (**B**) and (**D**) expand data for the Δ*dinJ* Δ*yafQ* Δ*Km*^*R*^/pCA24N-*yafQ* curves for (**A**) and (**C**), respectively. Data are averaged from three independent cultures, and one standard deviation is shown.

To address why YafQ reduces growth at low temperatures upon deletion of *dinJ* at 18°C, YafQ mRNA and toxicity were assayed in BW25113 wild-type and Δ*dinJ* Δ*Km*^*R*^ at 18°C vs. 37°C. Based on our qRT-PCR result, YafQ mRNA levels are unchanged in BW25113 wild-type and Δ*dinJ* Δ*Km*^*R*^ at both 18°C or 37°C (Table B in S1 File). We also explored YafQ toxicity at 37°C and 18°C and found that YafQ is more toxic at lower temperatures (Fig 4). Hence, the increase in YafQ toxicity upon *dinJ* deletion at 18°C is not due to changes in YafQ mRNA levels; however, it is unclear whether different amounts of YafQ protein contribute to this difference.

### Mlc suppresses YafQ toxicity

**Fig 5:**
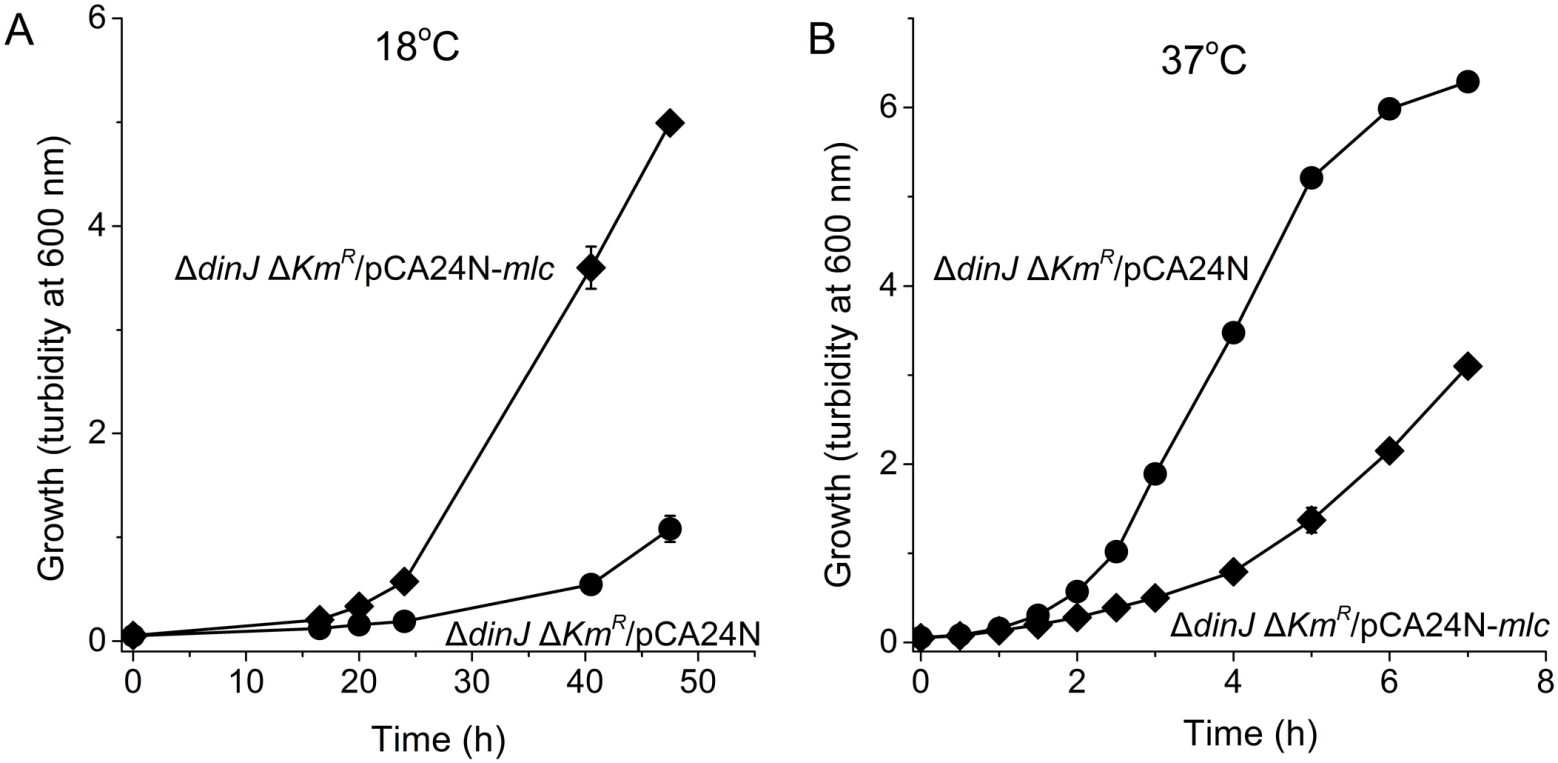
Mlc suppresses YafQ toxicity. Comparison of growth in LB medium for BW25113 *ΔdinJ ΔKm*^*R*^/pCA24N (●) and BW25113 *ΔdinJ ΔKm*^*R*^/pCA24N-*mlc* (♦) with 200 μM IPTG induction at 18°C (**A**) and 37°C (**B**). Data are averaged from three independent cultures, and one standard deviation is shown.

**Fig 6:**
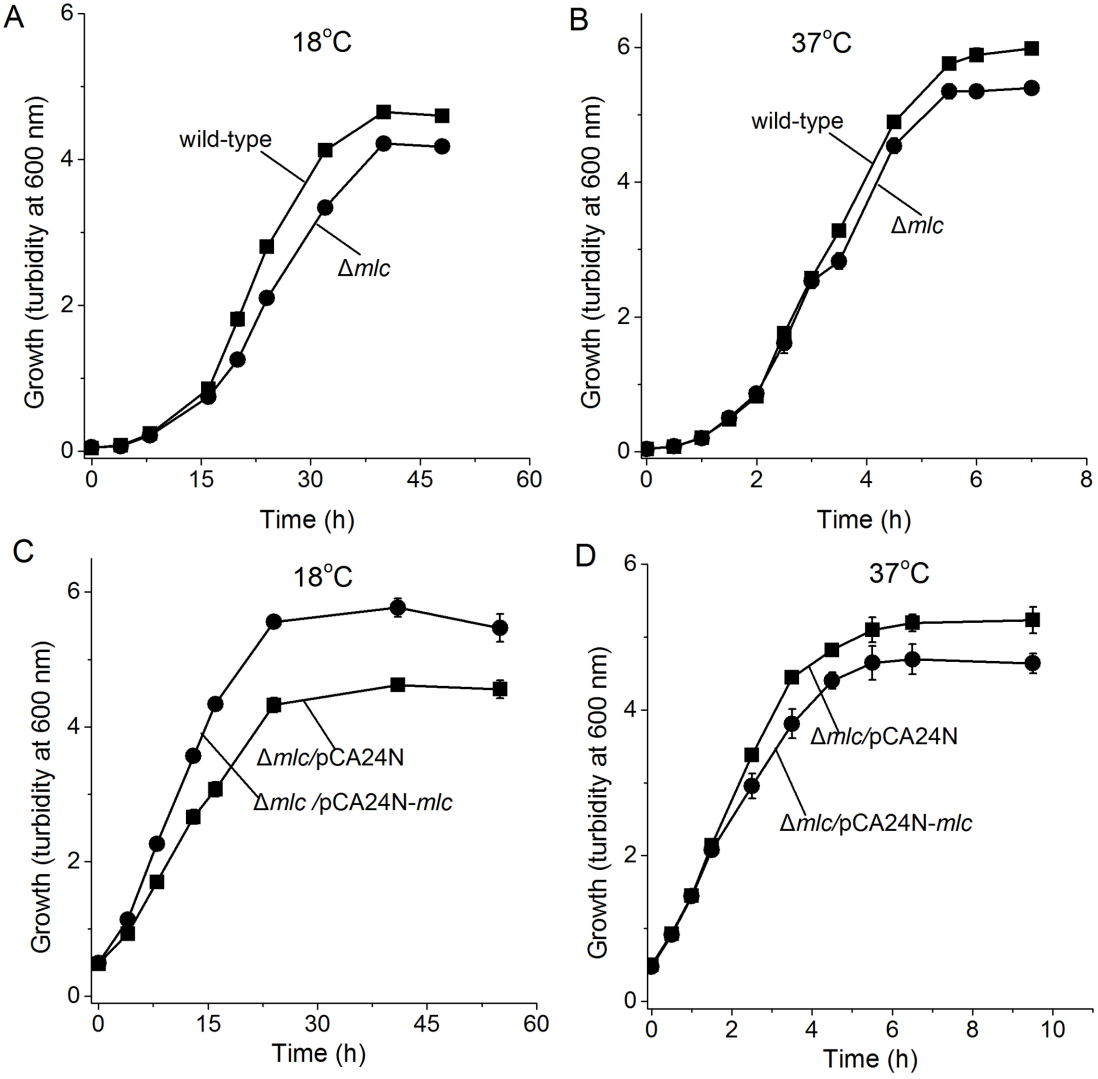
Δ*mlc* reduces growth at 18°C and 37°C. Comparison of growth in LB medium for BW25113 wild-type (■) and BW25113 Δ*mlc* (●) at 18°C (**A**) and 37°C (**B**). Comparison of growth in LB medium for BW25113 Δ*mlc*/pCA24N (■) and BW25113 Δ*mlc*/pCA24N-*mlc* (●) with 0.5 mM IPTG induction at 18°C (**C**) and 37°C (**D**). Data are averaged from three independent cultures and one standard deviation is shown.

Since YafQ was not toxic at 37°C in the absence of DinJ, we investigated whether a protein beyond DinJ masks YafQ toxicity. Hence, we selected for faster growth at 18°C (where YafQ was activated and slowed growth) after electroporation of a pooled ASKA library into Δ*dinJ* Δ*Km*^*R*^. After enrichment from six rounds of growth, four plasmids of the ASKA clone set were found that restored BW25113Δ*dinJ* Δ*Km*^*R*^ growth; sequencing revealed that the pCA24N plasmids encoded YhbU, SspA, and Mlc (Table 3). To avoid the influence of spontaneous mutations during the enrichment, the plasmids carrying *yhbU*, *sspA*, and *mlc* were re-electroporated into Δ*dinJ* Δ*Km*^*R*^, and Mlc was found to increase the growth of Δ*dinJ* Δ*Km*^*R*^ the most. Critically, Mlc increased growth in the absence of *dinJ* at 18°C while it decreased growth at 37°C (Fig 5); hence Mlc is toxic at 37°C. Corroborating these results, deletion of *mlc* in the wild-type strain decreased growth more at 18°C (Fig 6A and 6B), and this phenotype could be complemented by producing Mlc from plasmid pCA24N-*mlc* only at 18°C (Fig 6C and 6D). These results demonstrate that Mlc reduces YafQ toxicity in the absence of antitoxin DinJ but only at low temperatures.

**Table 3:** Proteins whose production from pCA24N increased growth at low temperatures in the Δ*dinJ* Δ*Km*^*R*^ strain as identified by screening the complete ASKA overexpression library.

Colony	Growth increase (%)	Protein	Function
1-3-57	32	YhbU	U32 peptidase family protein. *yhbUV* divergent operon promoter region binds FNR and *yhbUV* operon expression is activated by FNR
2-3-25	19	SspA	Stringent starvation protein A, phage P1 late gene activator; RNAP-associated acid-resistance protein; inactive glutathione S-transferase homolog
2-6-45	65	Mlc (DgsA)	Global transcriptional repressor; regulates pts operon expression at P0; required for anaerobic growth on glucosamine, binds *nagC* promoters; regulates *manX* and *malT*; makes large colonies; autorepressor
2-6-64	62		

### RpoS increases at 18°C when *dinJ* is deleted

Since DinJ reduces stationary phase sigma factor (RpoS) levels to affect the general stress response [29], we investigated if RpoS levels were altered by growth in the BW25113 *ΔdinJ ΔKm*^*R*^ mutant relative to the wild-type strain at 37°C and 18°C using a Western blot. As expected at 37°C [29], we found that DinJ inactivation results in roughly two fold more RpoS, and similar results were found at 18°C (Fig B in S1 File). Therefore, the quantity of RpoS is not the major reason for elevated toxicity of YafQ at 18°C.

## Discussion

In this study, we show clearly that inactivation of antitoxin DinJ results in slower growth and metabolism only at low temperatures. This reduction in growth is not from the interaction of DinJ and the stress response sigma factor RpoS [29] but instead from activation of toxin YafQ since both metabolism (Fig 1) and cell growth (Fig 2) were decreased only at this temperature and inactivation of YafQ (identified by a transposon search) restored growth at low temperatures (Fig 2). Therefore, the YafQ/DinJ TA system seems to be utilized primarily at low temperatures in a manner similar to the activation at low temperatures of toxin GraT of the type II GraT/GraA TA system of *Pseudomonas putida* [42]; toxin GraT inhibits ribosome assembly at low temperatures by interacting with the chaperone DnaK [55]. The other TA systems that are affected by temperature changes are the type I BsrG/SR4 TA system of *Bacillus subtilis* [41] in which toxin BsrG mRNA is degraded upon heat shock, and the single polypeptide toxin and antitoxin EzeT which only has toxicity at low temperatures due to changes in protein folding [43].

Since YafQ was not toxic at 37°C (in the absence of antitoxin DinJ, Fig 2B) but YafQ has activity both at 18°C and 37°C when it is overproduced from its non-native promoter (Fig 4), these results suggest that there is another protein (beyond DinJ) that inactivates YafQ at 37°C or that the primary target of YafQ is produced only at 18°C. Our attempt to find this suppressor/target, by overproducing all the *E*. *coli* proteins in a *dinJ* mutant, determined that the global transcription repressor Mlc suppresses YafQ toxicity effectively at 18°C (Fig 5); hence, the cell may use more than the toxin YafQ to regulate antitoxin activity or, more likely, overproducing one of the main targets of YafQ at 18°C, Mlc, prevents YafQ from slowing growth at low temperatures. In support of this hypothesis, Mlc has nine in-frame YafQ cleavage sites and no DinJ binding motifs so it is very likely the Mlc mRNA is degraded by YafQ. Also, Mlc is a ROK family transcriptional regulator that binds DNA via a helix-turn-helix motif and regulates glucose transport via its regulation of the phosphotranserase system [56]. In agreement with our hypothesis that Mlc may play a role regulating the activity of YafQ, *mlc* is induced during cold shock [57]. Therefore, the mechanism of slow growth only at low temperatures upon deleting antitoxin gene *dinJ* is activation of toxin YafQ (rather than DinJ interactions with other genes), and the ensuing toxicity due to activation of YafQ is probably cleavage of the cold-shock-related regulator Mlc.

In addition to the *yafQ* deletion, growth was restored to the *dinJ* mutant at 18°C by mutations in *rapA*, *hupA*, *gcvT*, *gcvP*, and *orfE* (Table 2). RapA, which shows the highest frequency of Tn insertion, 5 out of 12, stimulates RNA polymerase recycling in transcription [58]. Hence, Tn insertion in *rapA* perhaps lowered RNA polymerase recycling and thereby reduced *yafQ* transcription, resulting in a decrease of YafQ and increased growth.

HU is a major component of the bacterial nucleoid, composed of two non-identical but highly homologous subunits, HUα and HUβ [59, 60]. HUα is encoded by *hupA* (which shows the second highest frequency of Tn insertion, 2 out of 12). HUα is abundant during growth, while the heterodimeric αβ form predominates at the end of the exponential growth phase and remains as in the stationary phase[61]. HU is required for efficient expression of RpoS [62], and we have shown DinJ decreases RpoS levels [29] (Fig B in S1 File). Hence, Tn insertion in *hupA* could perhaps decrease *rpoS* translation and offset the increase in RpoS due to *dinJ* deletion, resulting in reduced of RpoS and therefore restoring the growth as the wild type strain.

RNase PH, encoded by *orfE*, could also play a role in structured RNA degradation [63]. YafQ is an endoribonuclease that associates with the ribosome through the 50S subunit and blocks translation elongation through mRNA cleavage at 5'-AAA-G/A-3' sequences. When *dinJ* is deleted, YafQ would be more toxic with higher endoribonuclease activity at 18°C, and the slow growth of the *ΔdinJ ΔKm*^*R*^ mutant is caused by the degradation of RNA in cell. Hence, an insertion of Tn in *orfE* perhaps slows the degradation structured RNA, which would restore growth of the *ΔdinJ ΔKm*^*R*^ mutant.

Proteins encoded by *gcvT* and *gcvP* belong to the glycine cleavage system, which is widely distributed in animals, plants, and bacteria [64] and catalyzes the oxidative cleavage of glycine to carbon dioxide, ammonia, and 5,10-methylenetetrahydrofolate as well as provides a secondary pathway for one-carbon biosynthesis [65–67]. It is not clear why growth of the *ΔdinJ ΔKm*^*R*^ mutant was restored when the glycine cleavage system is blocked by Tn mutation in *gcvT* and *gcvP*.

## Supporting Information

S1 File Fig A, Sequence analysis of BW25113 Δ*dinJ* Δ*Km*^*R*^. Fig B, Rpos levels detected by an anti-RpoS antibody. Table A, Oligonucleotides used for random amplification of transposon ends (RATE) PCR and qRT-PCR. Table B, Summary of qRT-PCR results.
(DOC)
