# Supplementary material for: Toxin YafQ Reduces Escherichia coli Growth at Low Temperatures
Source: PLoS One. 2016 Aug 24;11(8):e0161577. doi: 10.1371/journal.pone.0161577 (PMC4996492; doi:10.1371/journal.pone.0161577)
Supplement: S1 File — Fig A, Sequence analysis of BW25113 ΔdinJ ΔKmR. Fig B, Rpos levels detected by an anti-RpoS antibody. Table A, Oligonucleotides used for random amplification of transposon ends (RATE) PCR and qRT-PCR. Table B, Summary of qRT-PCR results. (DOC) [file pone.0161577.s001.doc]

**SUPPLEMENTARY INFORMATION**

**Toxin YafQ Reduces *Escherichia coli* Growth at Low Temperatures**

Yueju Zhao^1,2,3^, Michael J. McAnulty^3^, and Thomas K. Wood^3,4^

^1^Institute of Food Science and Technology, Chinese Academy of Agricultural Sciences and ^2^Key Laboratory of Agro-products Processing, Ministry of Agriculture, Beijing, P. R. China, 100193

^3^Department of Chemical Engineering and the ^4^Department of Biochemistry and Molecular Biology, Pennsylvania State University, University Park, Pennsylvania 16802-4400

*For correspondence. E-mail: [twood@engr.psu.edu](mailto:twood@engr.psu.edu); Tel.(+)1 814-863-4811; Fax (1) 814-865-7846

Running head: Toxin YafQ decreases growth at low temperature

**Table A. Oligonucleotides used for random amplification of transposon ends (RATE) PCR and qRT-PCR.** “F” indicates forward primers and “R” indicates reverse primers.

| **Primer name** | **Sequence (5’→3’)** |
| --- | --- |
| **RATE** | |
| Kan-2 FP-1 | ACCTACAACAAAGCTCTCATCAACC |
| Kan-2 RP-1 | GCAATGTAACATCAGAGATTTTGAG |
| Inv1 | ATGGCTCATAACACCCCTTGTATTA |
| Inv2 | GAACTTTTGCTGAGTTGAAGGATCA |
| **qRT-PCR** | |
| *rrsG*-RT-F | CAAGACCAAAGAGGGGGACC |
| *rrsG*-RT-R | GGACCGTGTCTCAGTTCCAG |
| *yafQ*-RT-F | ACTTTACCGCTTCCAGCTGTT |
| *yafQ*-RT-R | CGCGTGAGTTCCAGTTCTCT |

**Table B. Summary of qRT-PCR results.** The cycle number (C_t_) for each sample is indicated for the target gene *yafQ* as well as for the housekeeping gene, *rrsG*, which was used to normalize the data. Fold changes in transcription were calculated using :

2^-(C_t target, Δ_*_mqsRA_* - C_t_ *_rrsG_*_, Δ_*_mqsRA_*)/2^-(C_t target, wild-type_ - C_t_ *_rrsG_*_, wild-type_)

The specificity of the qRT-PCR products were verified by melting curve analysis . Means and standard deviations are indicated (*n* = 3).

| **Incubation temperature (^o^C)** | **Strain** | **C_t_ (target gene, *yafQ*)** | **C_t_ (housekeeping gene, *rrsG*)** | **Fold change** |
| --- | --- | --- | --- | --- |
| **37** | **BW25113 wild-type** | 20.85 ± 0.42 | 8.90 ± 0.38 | 1.2± 2.0 |
|  | **BW25113 Δ*dinJ* Δ*Km^R^*** | 20.28 ± 0.28 | 8.62 ± 0.37 |  |
| **18** | **BW25113 wild-type** | 20.71 ± 0.63 | 7.98 ± 0.08 | 1.9 ± 1.7 |
|  | **BW25113 Δ*dinJ* Δ*Km^R^*** | 18.98 ± 0.70 | 7.18 ± 0.49 |  |

**

**

**Fig. A. Sequence analysis of BW25113 Δ*dinJ* Δ*Km^R^*.**

**
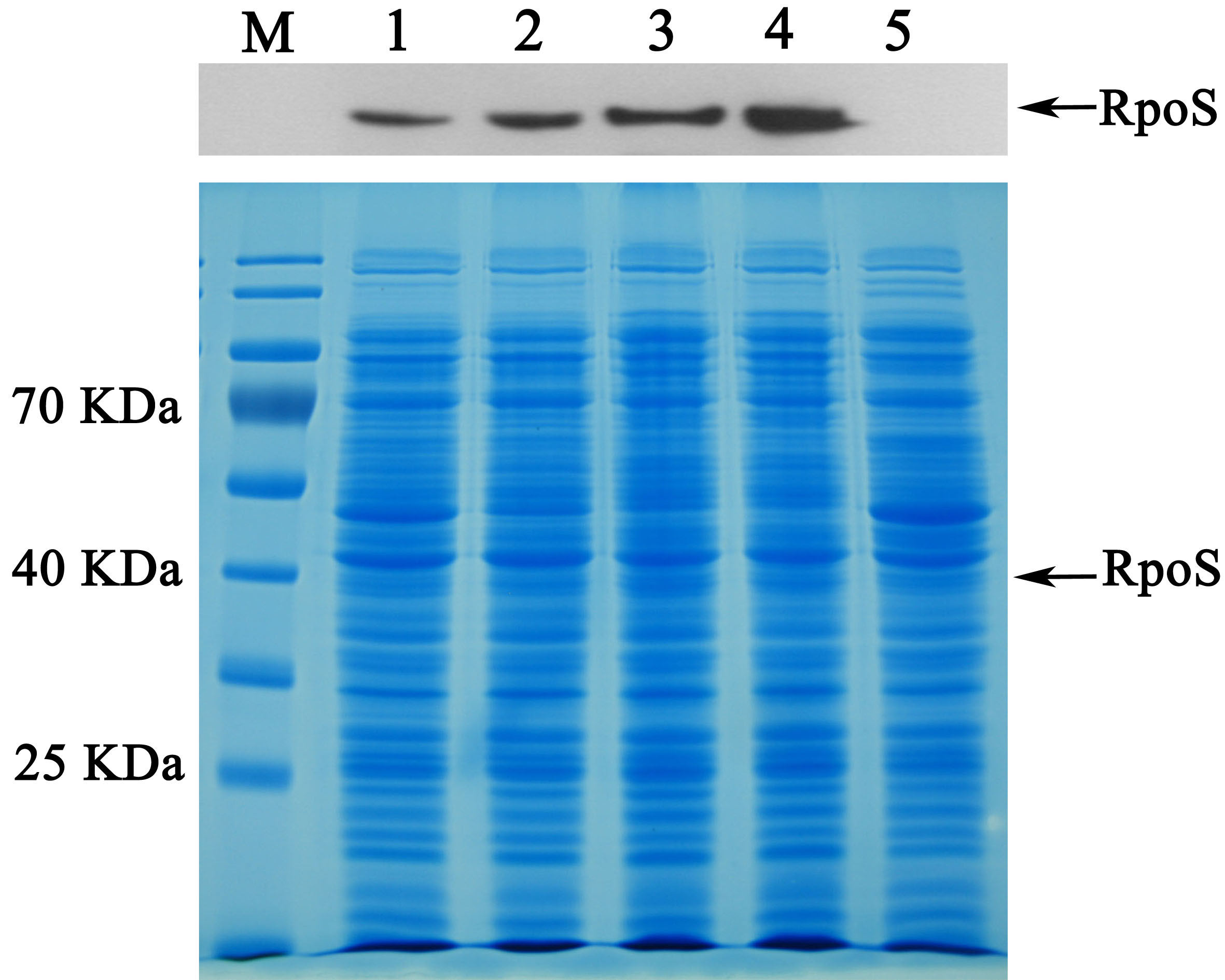
**

**Fig. B. Rpos levels detected by an anti-RpoS antibody.** Lane 1, BW25113 grown at 37 ºC, Lane 2, BW25113 Δ*dinJ*Δ*Km^R^* grown at 37 ºC, Lane 3, BW25113 grown at 18 ºC, Lane 4, BW25113Δ*dinJ*Δ*Km^R^* grown at 18 ºC, and Lane 5, BW25113Δ*rpoS*Δ*Km^R^* grown at 37 ºC (negative control). Cells grown for two hours at 37 ºC were harvested, and cells grown 16 hours at 18 ºC were harvested. 10 µl sample (4 µg/ µl, i.e. total protein content is 40 µg) was loaded in 12% SDS-PAGE.

**REFERENCES**

Pfaffl, M.W. (2001) A new mathematical model for relative quantification in real-time RT-PCR. *Nucleic Acids Res* **29**: e45.
